# Supplementary material for: Analysis of Genetic and MRI Changes, Blood Markers, and Risk Factors in a Twin Pair Discordant of Progressive Supranuclear Palsy
Source: Medicina (Kaunas). 2023 Sep 22;59(10):1696. doi: 10.3390/medicina59101696 (PMC10608279; doi:10.3390/medicina59101696)
Supplement: Supplementary file 1 [file medicina-59-01696-s001.zip › medicina-2600796-supplementary.pdf]

ST_1

APOE probe	FAM-CAGCTCCTCGGTCTGCTCTGGC-BHQ1
ε2-Forward	GCGGACATGGAGGACGTGT
ε2-Reverse	CCTGGTACACTGCCAGAGGCA
ε3-Forward	CGGACATGGAGGACGTGT
ε3-Reverse	CTGGTACACTGCCAGGCG
ε4-Forward	CGGACATGGAGGACGTGC
ε4-Reverse	CTGGTACACTGCCAGGCG
ACTB probe	HEX-TGCTGTCTGGCGGCACCACCATGTACC-BHQ1
ACTB-Forward	GACGTGGACATCCGCAAAGAC
ACTB-Reverse	CAGGTCAGCTCAGGCAGGAA
